# The Society of Thoracic Surgeons, The Society of Cardiovascular Anesthesiologists, and The American Society of ExtraCorporeal Technology: Clinical Practice Guidelines for Cardiopulmonary Bypass—Temperature Management during Cardiopulmonary Bypass

**Published:** 2015

**Authors:** Richard Engelman, Robert A. Baker, Donald S. Likosky, Alina Grigore, Timothy A. Dickinson, Linda Shore-Lesserson, John W. Hammon

**Affiliations:** Department of Surgery, Baystate Medical Center and Tufts University School of Medicine, Springfield, Massachusetts; Cardiac Surgery Research and Perfusion, Flinders University and Flinders Medical Centre, Adelaide, South Australia, Australia; Department of Cardiac Surgery, University of Michigan, Ann Arbor, Michigan; Department of Anesthesiology, University of Nevada, Las Vegas, Nevada; Clinical Performance Improvement, SpecialtyCare, Nashville, Tennessee; Department of Anesthesiology, Hofstra Northshore-Long Island Jewish School of Medicine, New Hyde Park, New York; and Department of Cardiothoracic Surgery, Wake Forest University School of Medicine, Winston-Salem, North Carolina

**Keywords:** cardiopulmonary bypass, perfusion, temperature management, cardiopulmonary bypass neurologic morbidity

## Abstract

To improve our understanding of the evidence-based literature supporting temperature management during adult cardiopulmonary bypass, The Society of Thoracic Surgeons, the Society of Cardiovascular Anesthesiology and the American Society of ExtraCorporeal Technology tasked the authors to conduct a review of the peer-reviewed literature, including 1) optimal site for temperature monitoring, 2) avoidance of hyperthermia, 3) peak cooling temperature gradient and cooling rate, and 4) peak warming temperature gradient and rewarming rate. Authors adopted the American College of Cardiology/American Heart Association method for development clinical practice guidelines, and arrived at the following recommendation.

## CLASS I RECOMMENDATIONS

1. **The oxygenator arterial outlet blood temperature is recommended to be used as a surrogate for cerebral temperature measurement during cardiopulmonary bypass (CPB). (Class I, Level C)**
2. **To monitor cerebral perfusate temperature during warming, it should be assumed that the oxygenator arterial outlet blood temperature underestimates cerebral perfusate temperature. (Class I, Level C)**
3. **Surgical teams should limit arterial outlet blood temperature to <37°C to avoid cerebral hyperthermia. (Class I, Level C)**
4. **Temperature gradients between the arterial outlet and venous inflow on the oxygenator during CPB cooling should not exceed 10°C to avoid generation of gaseous emboli. (Class I, Level C)**
5. **Temperature gradients between the arterial outlet and venous inflow on the oxygenator during CPB rewarming should not exceed 10°C to avoid outgassing when blood is returned to the patient. (Class I, Level C)**

## CLASS IIA RECOMMENDATIONS

1. **Pulmonary artery (PA) or nasopharyngeal temperature recording is reasonable for weaning and immediate postbypass temperature measurement. (Class IIa, Level C)**
2. **Rewarming when arterial blood outlet temperature ≥30° C:**
  - **To achieve the desired temperature for separation from bypass, it is reasonable to maintain a temperature gradient between arterial outlet temperature and the venous inflow of ≤4°C. (Class IIa, Level B)**
  - **To achieve the desired temperature for separation from bypass, it is reasonable to maintain a rewarming rate ≤.5°C/min. (Class IIa, Level B)**
3. **Rewarming when arterial blood outlet temperature <30^°^C: to achieve the desired temperature for separation from bypass, it is reasonable to maintain a maximal gradient of 10°C between arterial outlet temperature and venous inflow. (Class IIa, Level C)**

## NO RECOMMENDATION

**No recommendation for a guideline is provided concerning optimal temperature for weaning from CPB due to insufficient published evidence.**

Numerous strategies are currently invoked by perfusion teams to manage the requirements of cooling, temperature maintenance, and rewarming patients during cardiac surgical procedures. To date there have been very few evidence-based recommendations for the conduct of temperature management during perfusion. Although Bartels et al. (1) found no supporting evidence for an evidence-based guideline for managing the temperature gradient during CPB, Shann et al. (2) recommended that “limiting arterial line temperature to 37°C might be useful for avoiding cerebral hyperthermia,” including checking “coupled temperature” ports for all oxygenators for accuracy and calibration (Class IIa, Level B).

Owing to differences in interpreting the literature and the paucity of published guidelines in this clinical area, there is extensive variability in the conduct of managing perfusate temperature during CPB. A recent survey of perfusionists found that 1) in more than 90% of centers, mildly hypothermic perfusion of 32°C to 34°C is routinely used and 63% achieve that temperature without active cooling; 2) during CPB, the most common sites for measuring temperature are nasopharyngeal (NP, 84%), venous return (75%), arterial line (72%), bladder (41%), and rectum (28%); 3) 19% of centers reported routinely calibrating their in-line temperature probes; and 4) 44% of centers exceed the 37°C peak temperature limit for the arterial line temperature during rewarming (3). Although temperature management strategies are frequently reported in the literature, the rationale for these practices is often underreported or absent.

To improve our understanding of the evidence-based literature supporting temperature management, we conducted a review of the peer-reviewed literature, including 1) optimal site for temperature monitoring, 2) avoidance of hyperthermia, 3) peak cooling temperature gradient and cooling rate, and 4) peak warming temperature gradient and rewarming rate.

## MATERIALS AND METHODS

### Literature Search

We used a systematic search of Medical Subject Heading terms to identify peer-reviewed articles related to temperature management in the setting of adult CPB (Appendix). Candidate articles were published in PubMed between January 1, 2000 and March 31, 2014. Our search revealed 768 abstracts, all of which were reviewed in duplicate by independent reviewers, with 153 abstracts selected for full paper review. To be included, each paper had to report data on each of the following: 1) optimal site for temperature monitoring, 2) avoidance of hyperthermia, 3) peak cooling temperature gradient and cooling rate, and 4) peak warming temperature gradient and rewarming rate.

According to American College of Cardiology/American Heart Association (ACC/AHA) rules (Table 1), any reviewer could select an abstract for inclusion in a paper review, but at least two reviewers had to agree to exclude a paper (4). At the paper review stage, at least two reviewers had to agree to exclude a paper. These rules were incorporated into Guideliner reviewing software (www.guideliner.org/default.aspx, accessed May 20, 2014). Thirteen articles considered relevant to the topic by the authors were included to provide method, context, or additional supporting evidence for the recommendations.

**Table 1.** American College of Cardiology/American Heart Association: Classifications and level of evidence (4).

Classification	Clinical recommendation
Class I	Benefit >>> risk
	Conditions for which there is evidence and/or general agreement that a given procedure or treatment is useful and effective
Class II	Conditions for which there is conflicting evidence and/or a divergence of opinion about the usefulness/efficacy of a procedure or treatment. This is classified as IIa or IIb
Class IIa	Benefit >> risk
	Weight of evidence/opinion is in favor of usefulness/efficacy
Class IIb	Benefit ≥ risk
	Usefulness/efficacy is less well established by evidence/opinion
Class III	Risk > benefit
	Conditions for which there is evidence and/or general agreement that the procedure/treatment is not useful/effectiv and in some cases may be harmful. This is defined as: no benefit—procedure/test not helpful or treatment withou established proven benefit; harm—procedure/test/treatment leads to excess cost without benefit or is harmful
Level of evidence	Type of evidence
Level A	Evidence from multiple randomized trials or meta-analyses
Level B	Evidence from single randomized trial or nonrandomized studies
Level C	Evidence from expert opinion, case studies, or standard of care

## RESULTS

### Synthesizing the Evidence

**Figure 1.**
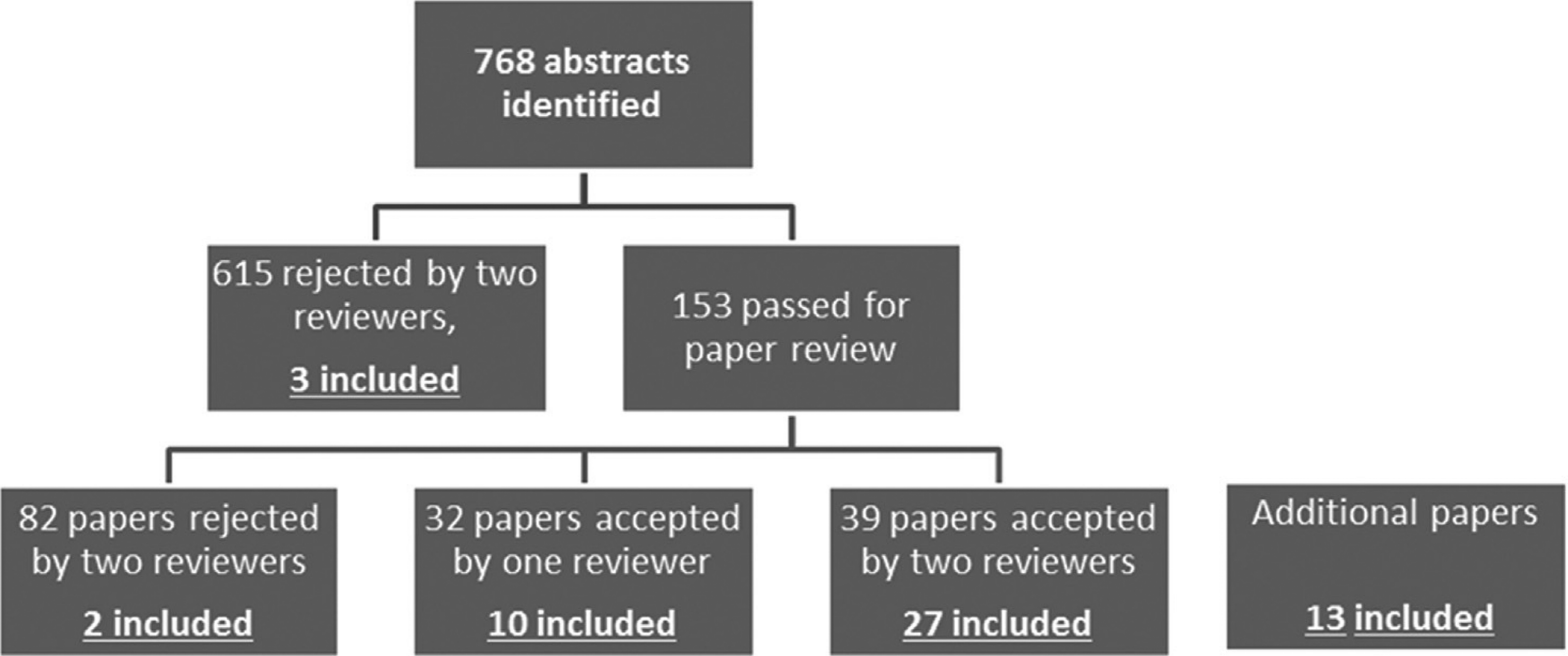
Flowchart illustrates the pathway of abstracts identified by the search strategy. Literature has been included in final selection (included) from various levels of the review process, including 13 papers not identified in the original search strategy.

Two reviewers rejected 615 abstracts based on a lack of relevance, leaving 153 abstracts for full paper review (Figure 1). Two panel members reviewed each paper, and 82 of these papers were found not to contribute to the topic by both reviewers, a further 32 had conflicting reviews and were individually resolved, and the final 39 were considered for inclusion in the guideline. Of the additional 13 articles relevant to the development of the Guideline 8 predated 2000.

### Optimal Site for Temperature Measurement

A number of sites for routine core and cerebral temperature management have been reported, including NP, tympanic membrane, bladder, esophagus, rectum, PA, jugular bulb (JB), arterial inflow, and venous return (3). A single, easily monitored, optimal core temperature site has not been reported, although the intravascular and intracorporeal location of a PA catheter probably renders this site the best access for core temperature recording. However, PA catheters are used infrequently in many centers, necessitating a different core temperature measurement site. The measurement of the JB temperature is recognized as being the best indicator of cerebral temperature (5,6), but it is not routinely used as a monitor and its accuracy depends on the optimal positioning of this invasive temperature probe (6).

Akata et al. (7), in a study to evaluate monitoring of brain temperature during deep hypothermic circulatory arrest, compared five sites with JB monitoring. The authors recommended the use of PA and NP locations for estimating cerebral temperature rather than forehead, bladder, and fingertip sites. Bladder and rectal temperatures are unreliable indicators of cerebral temperature during CPB and may be as much as 2°C to 4°C lower than the brain temperature when rewarming during CPB (6). Thus, use of NP temperature monitoring is preferable to bladder or rectal temperatures during rewarming to avoid potential cerebral hyperthermia (8–10).

Johnson et al. (11) highlighted, in a prospective study of 80 patients, the disparity in temperature measurement between the NP temperature and the arterial outlet temperature. In 2004, Kaukuntla et al. (12) demonstrated that commonly used core temperature monitoring sites (bladder, esophageal, and NP) were not accurate measures of cerebral temperature where JB temperature is used as the gold standard. The authors concluded that arterial outlet temperatures should be monitored to avoid cerebral hyperthermic inflow. Nussmeier et al. (6,8) demonstrated that arterial blood outlet temperature measurement provided the best correlate with JB temperature, followed by NP and esophageal locations. Nussmeier et al. (6) also demonstrated that all body sites overestimated JB temperature during cooling and underestimated JB temperature during rewarming, reporting that, “the arterial outlet site had the smallest average discrepancy of all temperature sites relative to the JB site.”

The accuracy of arterial blood outlet-coupled temperature ports has been reported in clinical and in vitro studies (13–16). These studies have consistently demonstrated variation between the temperature measured at the arterial outlet and a reference temperature measurement (a temperature probe placed distally within the circuit). Newland et al. (14) measured the arterial outlet temperature accuracy in four different oxygenators and reported that at 37° C, the arterial outlet temperature measurement underestimated the reference thermometer temperature by between .33°C and .67° C. These studies suggest that the maximum arterial blood outlet temperature target should be lower than 37°C to avoid perfusing the patient with blood temperatures higher than 37° C. Such corrections must be considered when using arterial outlet temperature as a surrogate site to monitor cerebral temperature.

After equilibration of core temperature after CPB, bladder temperature has been recommended as a good, noninvasive approach for monitoring core temperature (17). Bladder temperature probes have been shown to be reliable and correlate well with PA catheters in reflecting core body temperature in children (18). Khan et al. (19) demonstrated that tympanic membrane and axillary temperature measurements did not accurately reflect bladder temperature after CPB and suggested that clinical decisions should not be based on the former two temperature measurements.

#### Recommendation:

1. The oxygenator arterial outlet blood temperature is recommended to be used as a surrogate for cerebral temperature measurement during CPB. (Class I, Level C)
2. To accurately monitor cerebral perfusate temperature during warming, it should be assumed that the oxygenator arterial outlet blood temperature underestimates cerebral perfusate temperature. (Class I, Level C)
3. PA or NP temperature recording is reasonable for core temperature measurement. (Class IIa, Level C)

### Avoidance of Hyperthermia

Avoidance of cerebral hyperthermia in the setting of CPB has been promoted to avoid cerebral injury (10,12,20–22). Scheffer and Sanders (10) highlight that although the independent relationship between cerebral hyperthermia during CPB and neurologic injury has not been unequivocally proven, sufficient and compelling indirect evidence supports extreme caution. Shann et al. (2) applied evidence from the stroke literature to support a recommendation to limit the arterial blood outlet temperature to 37°C to avoid cerebral hyperthermia. Several reports have noted the association of increased cerebral temperature and rapid CPB rewarming with JB desaturation (23–25). Numerous authors have suggested that cerebral temperatures higher than 37°C should be avoided at any time during the rewarming phase of CPB and that this may be performed by careful monitoring and maintenance of arterial blood outlet temperatures lower than 37°C (15,20–22,26).

Grocott et al. (27) evaluated the effect of intraoperative temperature management in a randomized trial of 300 patients and found that the maximum postoperative temperature was weakly associated with increased cognitive dysfunction at 4–6 weeks. Hyperthermia has also been reported to influence other outcomes after cardiac operations. Groom et al. (28) reported an association with hyperthermia (defined as a peak core temperature >37.9° C) and an increased rate of mediastinitis. Newland et al. (29) reported in 2013 that an arterial outlet temperature exceeding 37°C during CPB is an independent predictor of acute kidney injury.

#### Recommendation:

Surgical teams should limit arterial outlet blood temperature to less than 37^°^C to avoid cerebral hyperthermia. (Class I, Level C)

### Peak Cooling Temperature Gradient and Cooling Rate

In 1997, Geissler et al. (30) demonstrated that temperature gradients (differential temperature between arterial outlet and venous inflow blood temperature) on cooling of more than 10°C were associated with gas emboli formation. They also reported no gaseous microemboli production when rapid cooling was used; however, this finding has not been reproduced. No contemporary literature has addressed this issue; however, a unique study in dogs in 1964 by Almond et al. (31) documented severe brain injury when an arterial-venous gradient of 20°C was used for cooling and significantly less injury when the cooling gradient was limited to 4° C. The authors proposed that rapid, high gradient cooling might not provide adequate cerebral hypothermia, preventing cerebral protection during induced cardiac arrest (the usual approach to cardiac operations in that era).

#### Recommendation:

Temperature gradients between the arterial outlet and venous inflow on the oxygenator during CPB cooling should not exceed 10^°^C to avoid generation of gaseous emboli. (Class I, Level C)

### Peak Warming Temperature Gradient and Rewarming Rate

The rate of rewarming during CPB and the temperature gradient to achieve this rewarming goal produces a conundrum for clinicians: the team must balance the deleterious effects of an extended duration of CPB and operative times with those that may be associated with rapid rewarming rates. A seminal issue has related to the potential for outgassing when warm blood is returned to the hypothermic patient, because dissolved gases come out of the blood when rapid rewarming takes place. Outgassing may be prevented by maintaining a maximal 10°C gradient between the arterial blood outlet and the venous inlet blood temperature (32). A number of studies have evaluated rewarming and temperature gradients, with a recommended gradient of not more than 10°C being most commonly reported (Table 2). Five clinical studies reported the influence of the rewarming rate (Table 3). Grigore et al. (45), in a cohort study evaluating cognitive performance of patients post-CPB, demonstrated a benefit associated with “slow” rewarming compared with “rapid” rewarming in the post-CPB cognitive performance of patients. In this study, slow rewarming (.49°C ± .17°C/min) was achieved with a maximal gradient of 2^°^C between the NP temperature and the CPB arterial blood outlet temperature, whereas rapid rewarming (.56°C ± .22°C/min) was achieved with a 4° Cto6°C maximal gradient. The slow rewarming group also avoided some of the exposure to excess hyperthermia and had temperatures in excess of 37°C for shorter periods. In this study, the gradient driving rewarming was between the NP temperature and the arterial blood outlet temperature. Borger and Rao (43) reported an observational study in which patients were evaluated for neurocognitive outcomes based on post hoc rewarming rates. The authors reported a decreased risk of neurocognitive decline with slow rewarming within one postoperative week, although this advantage was not present at 3 months.

**Table 2.** Rewarming rates and temperature gradients.*

First author	Study design	Number	Rewarming rate (° C/min)	Temperature gradient (° C)	Gradient between	Max arterial outlet	Monitoring site driving temperature management
Croughwell (25)	Prospective observational	133	NR	NR	NR	NR	NP
Cook (23)	Prospective observational	10	.56°C/min observational (estimated))^†^	10°C	H/E to blood	NR	NP, venous return
Geissler (30)	Animal	8	NR	0°C, 5°C, 10°C, 15°C, 20°C	Venous and arterial blood	NR	Thermodilution catheter placed in superior vena cava, arterial blood outlet (oxygenator)
Ginsberg (33)	Prospective, randomized	101	1°C every 3–5 minutes, achieved .2°C± .09°C, .19°C ± .07°C, .18°C ± .07°C	<10°C	H/E to venous	NR	PA catheter thermistor
Nathan (34)	Prospective, randomized	223	NR	NR	NR	NR	NP
Johnson (11)	Prospective, randomized	80	NR	NR	NR	38°C	NP
Schmid (35)				5°C	H/E to venous		
Lindholm (36)	Prospective, randomized	30	NR	10°C	H/E to venous	37–39°C	NP cooling, bladder rewarming
Ali (37)	Prospective, randomized	60	.33°C/min	NR	NR	NR	NR (“CPB temperature” perhaps arterial blood outlet)
Kaukuntla (12)	Prospective, randomized	60	NR	10°C	H/E to arterial inlet?	37.5°C	NP (hypothermic), bladder (normothermic)
Nussmeier (8)	Review	NA	.2° C/min	NR	NR	NR	NP, arterial line temperature?
Nussmeier (6)	Prospective observational	12/30	NR	NR	NR	37°C	JB, NP, esophageus, bladder, rectum, arterial blood outlet
Boodhwani (38)	Prospective, randomized	267	N/A	NR	NR	37.5°C/34.5°C	NP
Rasmussen (39)	Prospective, randomized	30	NR	10°C	H/E to venous	NR	NR
Hong (40)	Prospective observational	103	1°C every 5 minutes	NR	NR	37°C	NR
Akata (7)	Prospective observational	20	NR	Varying, as high as 20–25°C	JB to H/E	37.5°C	JB cooling, bladder rewarming
Boodhwani (41)	Prospective, randomized	223/267	NR	NR	NR	37.5°C/34.5°C	NP
Sahu(42)	Prospective, randomized	80	NR	2–4°C	H/E to blood	NR	NP
Newland (29)	Prospective observational	1393	<l°C/min	NR	NR	37.5°C/37°C	NP

* A description of reported trials (patient and animal) on rates of rewarming and arterial-venous temperature gradients (1992–2013).

† Calculated, not stated in original publication. H/E, heat exchanger; NR, not reported.

**Table 3.** Slow vs. fast rewarming: rates, gradients, and outcomes.^a^

			Slow Rewarming		Fast Rewarming					
First author	Study design	No.	Rate/duration	Gradient	Rate/duration	Gradient	Gradient between	Max arterial outlet	Monitoring site driving temperature management	Outcome measure
Cook 1996^23^	Prospective obsevational	10	.45°C/min (estimated)^b^	NR	.71°C/min (estimated)^b^	NR	H/E to blood	NR	NP	Cerebral venous oxygen desaturation less in slow group
Borger 2002^43^	Prospective observational	146	30 ± 9 min	NR	15 ± 4 min	NR		NR	NP	Neurocognitive function, benefit with slower rewarming rate
Diephuis 2002^44^	RCT	50	.24°C ± .08°C/min	5	.5°C ± .36° C/min	10	NP to H/E		NP	Cerebral pressure flow index unaffected by rewarming rate
Grigore 2002^45^	Prospective cohort blinded	165	.49°C ± .17°C/min	2	.56°C ± .22°C/min	4–6	NP and arterial outlet	NR	NP	Neurocognitive function, benefit with slower rewarming rate
Kawahara 2003^46^	RCT	100	.24°C/min	1–2	.48°C/min	4–5	Tympanic and arterial outlet	NR	Tympanic, PA	Jugular venous oxygen hemoglobin saturation, less decrease with slower rewarming rate
Saleh 2005^47^	RCT	30	.196°C ± .016°C/min	3	.34°C ± .027°C/min	7	NP to H/E		NP	Cardiac performance (cardiac index and peak velocity), blood lactate level, early homeostasis, ICU stays, improved with slower rewarming (medium rate, .248°C± .023°C)

*Abbreviations:* H/E = heat exchanger; ICU = Intensive care unit; NP = nasopharyngeal; NR = not reported; PA = pulmonary artery; RCT = randomized controlled trial.

a A description of reported trials (patients only) specific to slow vs. fast rewarming rates, arterial-venous gradients, and outcomes (1996–2005).

b Calculated, not stated in original publication.

Kawahara et al. (46), in a prospective randomized controlled trial (RCT) of 100 patients, demonstrated a reduction in the decrease in JB venous oxygen saturation with slow rewarming compared to rapid rewarming; however, they were unable to demonstrate any biochemical or neurologic benefits. They used similar temperature gradients as Grigore et al. (45), with a 1°C to 2°C difference between tympanic membrane and perfusate temperatures in the slow group (.24°C ± .09°C/min) and a 4°C to 5°C difference in the fast group (.48°C ± .09°C/min).

Sahu et al. (42), in a RCT in which the rewarming rate was tightly managed with a gradient of 2°C to 4°C, found similar reductions in JB venous oxygen saturation whether patients were rewarmed to 37°C or to 33° C. However, the authors reported less neurocognitive decline in patients rewarmed to only 33° C. The findings from this study suggest that exposure to cerebral hyperthermia may be associated with rapid rewarming and increased neurocognitive decline.

Diephuis et al. (44) evaluated the influence of fast (.5°C/min) and slow (.24°C/min) rewarming rates on cerebral blood flow, measured by transcranial Doppler, finding no significant effect of rewarming on the relation between pressure and cerebral blood flow. The authors used a maximal gradient of 10°C between the NP and water temperature in the fast group and a gradient of 5°C in the slow rewarming group.

In addition, an advantage associated with slow rewarming was supported by studies conducted in a pediatric population and in a swine model. Saleh and Barr (47) studied the effect of three rewarming strategies in pediatric patients and found that the slowest rewarming rate was associated with improved indices of cardiac function (improved cardiac index and decreased lactate production) but was disadvantaged by longer bypass times and time to reach core temperature targets. Alam et al. (48), in a swine model of lethal hemorrhage, investigated three rewarming rates from profound hypothermic arrest, slow (.25°C/min), medium (.5°C/min), and fast (1°C/min), and found a significant survival advantage in the group that was rewarmed at the medium rate compared with the fast rate. The group undergoing the slow rewarming rate had rewarming durations that were 1.7 and 2.1 times as long as those of the medium and fast groups, respectively, which may account for the worse outcomes in the slow group compared with those in the medium group.

The Ottawa Heart Institute published a series of reports discussing neurocognitive findings in two randomized coronary artery bypass grafting trials, each with more than 200 patients. In the first study, patients were randomized to rapid rewarming either to 37°C or to 34° C. The authors reported a significant early cognitive benefit noted in the relatively hypothermic patients (34,49). In this study arterial outlet temperatures were not reported nor were the absolute rewarming rates, potentially exposing the normothermic group to cerebral hyperthermia. Patients in the second randomized study were maintained at 37°C throughout the operation or maintained at 34°C without rewarming, thereby avoiding the rewarming process entirely. The authors found no differences in neurocognitive function between the two management strategies (38). They concluded that in the absence of active rewarming, cerebral hyperthermia was avoided. Although the conclusion of the two trials warrants the recommendation to avoid “rapid” rewarming to 37° C, the actual rate of rewarming in this “rapid” group was not reported. The data also suggest that normothermia itself is not associated with neurocognitive decline, but rather, that rapid rewarming to normothermia may lead to inadvertent cerebral hyperthermia.

Boodhwani et al. (41) later reported the influence of rewarming on renal function and concluded that the process of rewarming from 34°C to 37°C on CPB is associated with increased renal injury. This finding was supported by Newland et al. (29), who reported an arterial outlet temperature higher than 37^°^C is an independent predictor of acute kidney injury.

Multiple authors, including Kaukuntla et al. (50), suggest slow rewarming to avoid heterogeneous changes in brain temperature, and similarly, Murphy et al. (22) support gradual rewarming compared with rapid rewarming. Scheffer and Sanders (10) promote slow rewarming for a number of reasons including 1) avoidance of excess cerebral oxygen extraction and jugular venous desaturation; 2) improved maintenance of the relationship between cerebral blood flow and the cerebral metabolic rate of oxygen; and 3) increased time for better distribution of heat.

In a review paper comparing hypothermic with normothermic CPB, Grigore et al. (20) highlight the difficulty in assessing temperature management strategies as distinct from the confounder of rewarming rate. Only 4 of 15 RCTs reported information on rewarming rates. Cook (51) summarizes the case for slow rewarming most simply: its adoption increases the likelihood of preventing hyperthermia.

#### Recommendation:

1. Temperature gradients between the arterial outlet and venous inflow on the oxygenator during CPB rewarming should not exceed 10°C to avoid outgassing when warm blood is returned to the patient. (Class I, Level C)
2. Rewarming when arterial blood outlet temperature ≥30°C:
  - To achieve the desired temperature for separation from bypass, it is reasonable to maintain a temperature gradient between the arterial outlet and the venous inflow temperature of 4°C or less. (Class IIa, Level B)
  - To achieve the desired temperature for separation from bypass, it is reasonable to maintain a rewarming rate of .5°C/min or less. (Class IIa, Level B)
3. Rewarming when arterial blood outlet temperature is lower than 30°C: to achieve the desired temperature for separation from bypass, it is reasonable to maintain a maximal gradient of 10°C between the arterial outlet and venous inflow temperature. (Class IIa, Level C)

### Optimal Temperature for Weaning from CPB

There have been few compelling studies focused on the relationship between the temperature at the discontinuation of CPB and adverse sequelae. In the randomized trial by Nathan et al. (34) published in 2001, patients were rewarmed to 37°C or 34°C before CPB was discontinued. Cognitive function was assessed at three time points: early post-CPB, 3 months, and 5 years. An initial benefit was seen early post-CPB in the 34°C group (48% vs. 62% decline), but this was largely overcome by 3 months, and no statistically significant benefit was noted by 5 years (34,49,52).

Although it seems that much of the literature supports a hypothermic separation from CPB to protect cerebral metabolism, Insler et al. (53) reported outcomes in approximately 1600 coronary artery bypass patients who had core temperatures lower than 36°C on arrival in the intensive care unit (ICU). Adjusting for preoperative conditions, hypothermic patients had increased mortality, more transfusions, increased intubation time, and a longer ICU stay.

An additional consideration in hypothermia is the presence of post-CPB core temperature after drop, which is associated with reduced blood flow during CPB to muscle and viscera, and post-CPB heat transfer from the core to the periphery, resulting in core temperature depression (54). These are well-known phenomena and have associated morbidities themselves (55). Given the paucity of data and conflicting literature on weaning temperature and outcome after CPB, no specific weaning temperature is recommended. In the absence of a specific recommendation, the choice of temperature for weaning from CPB should be balanced between avoidance of cerebral hyperthermia and minimization of coagulopathy and transfusion. Post-CPB surface heating can be used in the postoperative cardiac surgical patient, but these techniques are not in the purview of this guideline.

***No Recommendation:*** No specific recommendation for an optimal temperature for weaning from CPB may be made due to inconsistent published evidence.

## SUMMARY

The importance of accurately recording and reporting temperature management during CPB cannot be overstated. Unfortunately, many published articles fail to document temperature management strategies during and after CPB.

Temperature management during CPB remains controversial, with gaps in our knowledge concerning a variety of aspects of temperature management. The Institute of Medicine has identified the need to incorporate the best clinical evidence into practice. Importantly, these guidelines challenge the cardiac surgical community to conduct research to address these gaps in knowledge. The summary of recommendations for temperature management during CPB is shown in Table 4.

**Table 4.** Recommendations for temperature management during cardiopulmonary bypass.*

Recommendation	Classification
Optimal site for temperature measurement	
The oxygenator arterial outlet blood temperature is recommended to be used as a surrogate for cerebral temperature measurement during CPB	Class I, Level C
To accurately monitor cerebral perfusate temperature during warming, it should be assumed that the oxygenator arterial outlet blood temperature underestimates cerebral perfusate temperature	Class I, Level C
PA catheter or NP temperature recording is reasonable for weaning and immediate postbypass temperature measurement	Class IIa, Level C
Avoidance of hyperthermia	
Surgical teams should limit arterial outlet blood temperature to <37°C to avoid cerebral hyperthermia	Class I, Level C
Peak cooling temperature gradient and cooling rate	
Temperature gradients from the arterial outlet and venous inflow on the oxygenator during CPB cooling should not exceed 10°C to avoid generation of gaseous emboli	Class 1, Level C
Peak warming temperature gradient and rewarming rate	
Temperature gradients from the arterial outlet and venous inflow on the oxygenator during CPB rewarming should not exceed 10°C to avoid outgassing when warm blood is returned to the patient	Class I, Level C
Rewarming when arterial blood outlet temperature >30° C	
To achieve the desired temperature for separation from bypass, it is reasonable to maintain a temperature gradient between arterial outlet temperature and the venous inflow of ≤4° C	Class IIa, Level B
To achieve the desired temperature for separation from bypass, it is reasonable to maintain a rewarming rate of ≤.5°C/min	Class IIa, Level B
Rewarming when arterial blood outlet temperature is <30° C	
To achieve the desired temperature for separation from bypass, it is reasonable to maintain a maximal gradient of 10°C between arterial outlet temperature and venous inflow	Class IIa, Level C

* The summary of the recommendations for temperature management during CPB.

## APPENDIX

Medical Subject Heading (MeSH) terms used to identify peer-reviewed articles related to temperature management in the setting of adult cardiopulmonary bypass:

(“cardiopulmonary bypass” [MeSH Terms] *or* perfusion [TIAB]) *and* (“body temperature” [MeSH Terms] or “body temperature regulation”[MeSH Terms] or “rewarming” [MeSH Terms]) and (“humans” [MeSH Terms] and English [lang] *and* “adult” [MeSH Terms])

((“cardiopulmonary bypass” [MeSH Terms] *or* cardiopulmonary bypass [TIAB]) *and* (“hypothermia, induced/instrumentation” [MAJR:noexp] or “hypothermia, induced/methods” [MAJR:noexp] or “body temperature” [MeSH Terms] *or* “body temperature regulation” [MeSH Terms] or “rewarming”[MeSH Terms]) *and* (“humans”[MeSH Terms] *and* English [lang])) *not* ((“cardiopulmonary bypass”[MeSH Terms] or perfusion [TIAB]) *and* (“body temperature” [MeSH Terms] *or* “body temperature regulation” [MeSH Terms] *or* “rewarming” [MeSH Terms]) *and *(“humans” [MeSH Terms] *and* English [lang] *and* “adult” [MeSH Terms])).
